# High-Throughput Sequencing Haplotype Analysis Indicates in *LRRK2* Gene a Potential Risk Factor for Endemic Parkinsonism in Southeastern Moravia, Czech Republic

**DOI:** 10.3390/life12010121

**Published:** 2022-01-14

**Authors:** Kristyna Kolarikova, Radek Vodicka, Radek Vrtel, Julia Stellmachova, Martin Prochazka, Katerina Mensikova, Tereza Bartonikova, Tomas Furst, Petr Kanovsky, Jan Geryk

**Affiliations:** 1 Department of Medical Genetics, Faculty of Medicine and Dentistry, Palacky University, 779 00 Olomouc, Czech Republic; kristyna.kolarikova@fnol.cz (K.K.); radek.vrtel@fnol.cz (R.V.); julia.stellmachova@fnol.cz (J.S.); martin.prochazka@fnol.cz (M.P.); 2 Department of Medical Genetics, University Hospital Olomouc, 779 00 Olomouc, Czech Republic; 3 Department of Neurology, Faculty of Medicine and Dentistry, Palacky University, 779 00 Olomouc, Czech Republic; katerina.mensikova@fnol.cz (K.M.); tereza.bartonikova@fnol.cz (T.B.); petr.kanovsky@fnol.cz (P.K.); 4 Department of Neurology, University Hospital Olomouc, 779 00 Olomouc, Czech Republic; 5 Department of Mathematical Analysis and Applications of Mathematics, Faculty of Science, Palacky University, 779 00 Olomouc, Czech Republic; tomas.furst@seznam.cz; 6 First Faculty of Medicine, Institute of Biology and Medical Genetics, Charles University and General University Hospital in Prague, 128 00 Prague, Czech Republic; jan.geryk@lfmotol.cuni.cz; 7 Second Faculty of Medicine, Institute of Biology and Medical Genetics, Charles University, 150 06 Prague, Czech Republic

**Keywords:** Parkinson’s disease, atypical Parkinson syndrome, *LRRK2* gene, haplotype, high-throughput sequencing

## Abstract

Parkinson’s disease and parkinsonism are relatively common neurodegenerative disorders. This study aimed to assess potential genetic risk factors of haplotypes in genes associated with parkinsonism in a population in which endemic parkinsonism and atypical parkinsonism have recently been found. The genes *ADH1C, EIF4G1, FBXO7, GBA, GIGYF2, HTRA2, LRRK2, MAPT, PARK2, PARK7, PINK1 PLA2G6, SNCA, UCHL1,* and *VPS35* were analyzed in 62 patients (P) and 69 age-matched controls from the researched area (C1). Variants were acquired by high-throughput sequencing using Ion Torrent workflow. As another set of controls, the whole genome sequencing data from 100 healthy non-related individuals from the Czech population were used (C2); the results were also compared with the Genome Project data (C3). We observed shared findings of four intron (rs11564187, rs36220738, rs200829235, and rs3789329) and one exon variant (rs33995883) in the *LRRK2* gene in six patients. A comparison of the C1–C3 groups revealed significant differences in haplotype frequencies between ratio of 2.09 for C1, 1.65 for C2, and 6.3 for C3, and odds ratios of 13.15 for C1, 2.58 for C2, and 7.6 for C3 were estimated. The co-occurrence of five variants in the *LRRK2* gene (very probably in haplotype) could be an important potential risk factor for the development of parkinsonism, even outside the recently described pedigrees in the researched area where endemic parkinsonism is present.

## 1. Introduction

Parkinson disease (PD) is a very frequent neurodegenerative disease which affects 1–2% of individuals in the population over 65 years of age and about 4% in the population over the age of 85 years [1,2].

The disorder is characteristic by the depletion of dopaminergic neurons in substantia nigra pars compacta in the midbrain and it is associated with the following most common symptoms: bradykinesia, muscle rigidity, and postural instability [3]. The major risk factors which contribute to PD development are age, environmental factors [4,5], and genetic background. The familial form of PD (about 5%) is caused by causal variant with high penetrance. The variants with minor effect and environmental factors could contribute to sporadic form [6].

Genes associated with parkinsonism have been studied for many years. The gene *SNCA* (OMIM 163890) is the first described gene responsible for PD [7]. Thanks to progress of molecular methods, additional PD-associated genes were identified even with dominant or recessive inheritance: *UCHL1* (OMIM 191342) [8,9,10], *LRRK2* (OMIM 609007) [11,12,13,14,15], *GIGYF2* (OMIM 612003) [16], *HTRA2* (OMIM 606441) [17], *VPS35* (OMIM 601501) [18,19], *EIF4G1* (OMIM 600495) [20], *ADH1C* (OMIM 103730) [21], *GBA* (OMIM 606463) [22,23], *MAPT* (OMIM 157140) [24], *PINK1* (OMIM 608309) [25], *PARK7* (OMIM 602533) [26], *PARK2* (OMIM 600116) [27], *PLA2G6* (OMIM 603604) [28], and *FBXO7* (OMIM 605648) [29].

The high prevalence of neurodegenerative parkinsonism has been described in some particular small, isolated, and endemic populations [30,31,32,33,34]. In such populations, higher contribution of genetic factors can be hypothesized.

In the Czech Republic, Mensikova et al. found a higher prevalence of parkinsonism in the southeastern Moravian region known as Hornacko [Upper Lands]; the disease showed a clear inheritance pattern, expressed in three large pedigrees [35,36]. Hornacko is predominantly hilly in the White Carpathian Mountains and borders Slovakia (formerly part of Hungary) and the Moravian Dolnacko (Lower Lands). The original inhabitants were attacked by Hungarian and Turkish raids in the 1600s and 1700s; in the mid-1800s, the region was colonized by newcomers from Silesia, western Slovakia, and Burgenland. These people formed a small isolated population with distinctive local traditions and religion.

In our epidemiological study, we managed to obtain DNA samples from patients and healthy controls from the region [37]. Thus, the opportunity of molecular-genetic analyses has arisen.

The aim of this study was to assess the co-occurrence of rare variants, possibly in haplotype, in the set of genes associated with parkinsonism (*ADH1C, EIF4G1, FBXO7, GBA, GIGYF2, HTRA2, LRRK2, MAPT, PARK2, PARK7, PINK1, PLA2G6, SNCA, UCHL1,* and *VPS35*) in the parkinsonian patients and controls from the Hornacko region [36,37,38]. To compare the population frequency of potential haplotype in patients, three different types of controls were used: (i) controls from the “Hornacko”, (ii) healthy non-related individuals, and (iii) sequencing data from The 1000 Genome Project data.

## 2. Materials and Methods

The study was written approved by the ethics committee of the Palacky University and University Hospital Olomouc, Czech Republic (IRB number 22-16985S;78_21). All experiments were performed in accordance with guidelines and regulations released by Palacky University and University Hospital Olomouc, Czech Republic. The patients were informed in detail about the study and they all signed informed consent.

The study involved 62 parkinsonian patients and 69 age-matched healthy controls from the same region (C1). The controls cohort includes 42 women and 27 men and their average age was over 70 years old. Demographic data are shown in Table 1.

The diagnosis of neurodegenerative parkinsonism was made at the Department of Neurology in University Hospital Olomouc, Czech Republic; the diagnostic process has been described in detail elsewhere [37,38]. The 69 age-matched healthy controls originating from and living in the Hornacko region were recruited through the general practitioner’s offices. The DNA was isolated in all patients and controls from peripheral blood with an isolation kit (QIAamp DNA Mini Kit from Qiagen). High-throughput sequencing was performed using the Personal Genome Machine (PGM) from Ion Torrent technology in the genes *ADH1C, ATP13A2, EIF4G1, FBXO7, GBA + GBAP1, GIGYF2, HTRA2, LRRK2, MAPT, PARK2, PARK7, PINK1, PLA2G6, SNCA, UCHL1,* and *VPS35*; performed target sequencing was described in detail in our previous study [37].

All variants found were filtered out by a minor allele frequency (MAF) of 0.05. A matrix was created in which the reference allele is marked as 0, heterozygote as 1, and homozygote as 2.

Using Microsoft Excel software, the data were filtered with respect to two criteria:

(1) Co-occurrence of two or more variants in a particular gene;
(2) Variants occurring mostly in the parkinsonian patients.

The detected variants were then verified and confirmed by Sanger sequencing. SIFT, MutationTaster, DANN, and PolyPhen-2 prediction tools were used to evaluate missense variants. The PhyloP algorithm was used to assess the phylogenetic conservation. Alternative splicing analysis was performed using NetGene2 and Human Splicing Finder 3.1 for the intron variants.

To evaluate (and exclude) possible consanguinity, the HVR1 mitochondrial region in six random patients and 15 Y-STR markers in five random male patients were analyzed using the AmpFLSTR™ Yfiler™ PCR Amplification Kit from Thermo Fisher Scientific.

To get a larger set of controls (C2), we used whole genome sequencing (WGS) data in 100 healthy nonrelated individuals from the Czech population (100CZG), originating from the Institute of Biology and Medical Genetics, Charles University, Prague, Czech Republic. The healthy individuals ranged in age from 23 to 60 years old. Individual DNA was used for WGS on NovaSeq 6000 (Illumina). The FASTQ data were processed by the generic data pre-processing pipeline published by the Broad Institute (https://github.com/gatk-workflows/gatk4-data-processing, acceesed on 21 December 2021). This pipeline aligns FASTQ data to hg38 genome build, performs base recalibration, and produces analysis-ready BAM files.

As another representative set of controls (C3), allelic frequency data from 2516 samples from the 1000 Genome Project (1000GP) were used for population-genetic comparison.

### Statistical Assessment

The demographic and clinical characteristics of the patients were presented as mean values ± standard deviation Fisher’s exact test or chi-square test was used to assess statistical differences in haplotype frequency, where appropriate. Relative risk (RR) and odds ratio (OR) were calculated. Differences were considered significant at a *p* < 0.05. All *p*-values were obtained from two-tailed tests, and all the analyses were performed in the MATLAB environment, MATLAB Version 7.5.0.342 (R2007b), Statistics Toolbox, Version 6.1.

## 3. Results

No co-occurrence of two or more rare variants in the genes *ADH1C, EIF4G1, FBXO7, GBA + GBAP1, GIGYF2, HTRA2, MAPT, PARK2, PARK7, PINK1, PLA2G6, SNCA, UCHL1*, or *VPS35* in any of the patients was revealed. The shared occurrence of four rare intron and one exon variants in the *LRRK2* gene was found in ten patients (Nr. 3, 17, 22, 23, 24, 26, 7 (11/32), 8 (19/32), 9 (21/32), and 10 (3/32)) (Table 2).

Patient clinical data are described in Table 3 in more detail. Possible patient consanguinity was excluded using HVR1 and Y-STR markers (Supplementary Tables S1 and S2).

The exon variant rs33995883 was benign according to SIFT; Polyphen-2 evaluated it as pathogenic, as did MutationTaster and DANN. Three intron variants (rs11564187, rs36220738, and rs3789329) do not affect alternative splicing according to NetGene2; deleting rs200829235 resulted in a possible break of the splice site. Substitution rs33995883 creates the acceptor of the splice site (according to NetGene2).

The intron variant rs11564187 led to the creation of a new donor site (based on HSF Matrices), according to Human Splicing Finder 3.1. A difference between mutant and reference sequences was not predicted for variant rs36220738. The variant rs200829235 resulted in the activation of an exonic cryptic donor site and the potential alteration of mRNA splicing (based on HSF Matrices). The variant rs33995883 led to the activation of an exonic cryptic donor site and the potential alteration of splicing (based on HSF Matrices). No significant splicing motif alteration was detected in rs3789329.

A difference between haplotype frequency between the patients and controls were found (C1, *p* = 0.0031, RR 2.09, OR 13.15 Table S3; C2, *p* = 0.11, RR 1.65, OR 2.58, Table S4; C3, *p* < 0.0001, RR 6.3, OR 7.6, Table S5); however, the difference in haplotype frequency between the patients and C2 reached just a borderline statistical significance. Relative risks and odd ratios are summarized in Table S6.

## 4. Discussion

Many key factors have been identified in the past, which are putatively important in the development of neurodegenerative parkinsonism, including genetic predisposition, environmental risk factors, and lifestyle. In the term of genetics, rare variants in the *LRRK2* gene are the most common cause of hereditary parkinsonism. The *LRRK2* gene is associated with autosomal-dominant, late-onset familial “Parkinson’s disease” (PD). However, this hereditary disease may lack a seminal pathological hallmark of PD—the Lewy-body pathology—so the term “Parkinson’s disease” in the disease designation should probably be avoided [12,39,40,41,42,43].

The *LRRK2* gene is located in the 12q12 chromosome. The *LRRK2* gene contains 56 exons (https://www.ncbi.nlm.nih.gov/gene/120892 (accessed on 21 December 2021)). *LRRK2* is a member of the leucine-rich repeat kinase family with kinase and GTPase activities, and it encodes multiple domain proteins. The gene influences mitochondrial functions, autophagy [44], and signaling transmission [45]. *LRRK2* is expressed in neuronal cells as well as in immune system cells [46,47]. Among all the variants in the *LRRK2* gene, substitution p.G2019S is the most common variant in PD patients (1% of sporadic PD and 4% of familial PD) [11,48] and its frequency is more common in North African and Ashkenazi Jewish patients [49,50]. Another variant in the *LRRK2* gene, p.N2081D, was described as a risk factor for Crohn’s disease [51]. The presence of multiple minor variants (p.S16478, p.R1628P, and p.G2385R) in the *LRRK2* gene is usually correlated with a lower age at the Parkinson´s disease onset—52.6 (±12.3) years—as compared to patients without these risk variants—62.5 years (±10.5) [52]. A protective effect of the *LRRK2* variants p.N551K, p.R1398H, and p.K1423K was proposed [53].

Paisán-Ruiz, et al. (2009) performed a genome-wide association analysis focused on low frequency alleles that were found together in the *LRRK2* gene; the study identified 84 single nucleotide polymorphisms (SNP) for 49 linkage disequilibrium (LD) blocks. These loci were in strong LD [54]. In our study, we have found some of the same *LRRK2* variants as Paisán-Ruiz et al. (2009b) (rs11564187, rs33995883, and rs3789329) [55], but they all were assigned to another block.

Haplotypes associated with PD were studied in the *MAPT* gene. The study by Setó-Salvia (2011) reported a higher occurrence of the H1 haplotype (G3010A and A2706G or C7028T) in patients than in controls (*p* = 0.001) [56]. The rare sub-haplotype of the H1 haplotype, H1p, was more common in patients with another type of Lewy body disease, Parkinson’s disease dementia–PDD [11]. Our previous studies did not find any pathogenic “founder” mutation in the researched population of Hornacko; however, we have found variants with possible smaller effects (*MAPT*, *HTRA2*, and *LRRK2*) [36,37]. When assessing the degree of effect of a given genotype, it is also very important to keep in mind the common occurrence of variants in a particular haplotype.

In this study, in the *LRRK2* gene, the occurrence of intron variants almost exclusively together with the rarest variant (p.Asn2018Asp)–cis binding was detected (based on 1000GP data and 100CZG data). We analyzed the presence of common 20 SNPs in the patients and three randomly selected control groups. In the patients, it was possible to assign identical haplotypes in all 20 SNPs; in the controls, the haplotype differentiated the controls from the patients and even from each other (Table S7). Therefore, we strongly suspect that the variants found are in the haplotype. The exon variant p.Asn2018Asp is located in the kinase domain, which is essential for *LRRK2*-induced neuronal toxicity [13,57,58].

Furthermore, we also looked for the co-occurrence of the haplotype in another population of 1000GP (The 1000 Genomes Project Consortium, 2015) [59]. The frequencies of the haplotype in different populations are compared in Table 4.

Most of the populations with the haplotype come from Europe or have European ancestors: Tuscans in Italy, Iberians in Spain, Finnish British in England and Scotland, Utah residents with Northern and Western European ancestry, Colombians in Medellin, and Gujarati from India. It could be explained by historical positive selection pressure (physical fitness at a younger age, resistance to climate change and infectious diseases, etc.). Because of the later onset of the disease, the risk factor for PD did not have to be manifested in everybody and the haplotype could, thus, remain conserved.

According to Greggio and Cookson (2009), missense variant p. Gly2019Ser, located in the kinase domain, leads to the increase of kinase activity and contributes to PD pathogenesis [60]. Inhibition of kinase activity may be considered as a potential therapeutic target [57,61]. Abnormal kinase activity of LRRK2 may result in abnormal phosphorylation of proteins (SNCA and MAPT) and it leads to aggregation and cell death [12]. We can assume that the variant has “some”, undoubtedly rather weak, impact on the development of neurodegenerative parkinsonism. Whether this is also true in the Hornacko population remains to be assessed in further studies involving all inhabitants of the researched area, i.e., almost 9000 people.

## 5. Conclusions

The co-occurrence of five variants in *LRRK2* gene was more frequent in patients with parkinsonism in Hornacko region. Intron variants occur almost exclusively with the rarest exon variant. *LRRK2* gene rate variants in haplotype might be a potential risk factor for endemic parkinsonism.

## Figures and Tables

**Table 1:** Demographic data of patients with neurodegenerative parkinsonism.

	No. of Subjects; F/M	Mean Age F	Mean Age M	Mean Age at Disease Onset F	Mean Age at Disease Onset M
Patients	30/32	71.8 (±12)	75.0 (±11)	61.5 (±9.5)	62.7 (±10)
Control subjects	42/27	75.09 (±9.65)	73.2 (±10.04)	-	-

No.: number, M: male, F: female; standard deviation is given in brackets.

**Table 2:** Shared variants in *LRRK2* gene (transcript NM_198578.3) in patients no. 1 (3), 2 (17), 3 (22), 4 (23), 5 (24), 6 (26), 7 (11/32), 8 (19/32), 9 (21/32), and 10 (3/32).

Variant	Coordinate	rs ID	EURMAF	Prediction NetGene2	Prediction Human Splicing Finder	Prediction SIFT/PolyPhen-2/ Mutationtaster/DANN	PhyloP
c.572-82A > G	12:40634203	rs11564187	0.02126	—	creation of new donor site	—	0.1
c.2242-22C > T	12:40677655	rs36220738	0.02081	—	—	—	−0.8
c.4317 + 12delT	12:40703047	rs200829235	0.02146	possible break of splicing site	potential alteration of mRNA splicing	—	1.5
c.6241A > G	12:40740686	rs33995883	0.01917	creation of acceptor splice site	activation of an exonic cryptic donor site	0.081/0.983/0.9874/0.9981	7.1
c.7391-44T > C	12:40760764	rs3789329	0.02297	—	—	—	−0.5

No: number of patient: the first number designates the patient in this study and the second number designates the patient within the whole study group; the variant is described according to coding sequence HGVS nomenclature; coordinate is the location in the genome related to reference genome hg19; rs ID is the identifier of the variant; EURMAF is the minor allele frequency in the European population; in silico prediction tools NetGene2 and Human Splicing Finder are used to predict that possible impact on hnRNA splicing; SIFT, PolyPhen-2, MutationTaster, and DANN are in silico prediction tools for evaluating the variant on the protein function; PhyloP score evaluates the phylogenetic conservation of a particular site in a genome.

**Table 3:** Summary of the patient clinical data.

Pat. No.	Gender/Age	Age at the Disease Onset	Clinical Phenotype	Clinical Signs Present at Examination
1 (3)	M/75	66	MSA-C	Atypical parkinsonian syndrome with static tremor of upper limbs, bilateral neocerebellar symptoms, rigidity, and postural instability; without any cognitive or executive dysfunction.
2 (17)	M/79	68	IPD	Typical rigidity-dominant PD with rigidity, bradykinesia, and rest tremor of upper limbs, advanced stage with the presence of late motor complications (patient treated with continuous intrajejunal infusion of L-DOPA gel).
3 (22)	M/66	45	Tremulous form of atypical parkinsonism with orthostatic hypotension	Static and rest tremor of upper limbs, static tremor of the head, and axial propriospinal myoclonus, without any cognitive or executive dysfunction.
4 (23)	M/61	49	IPD	Typical tremor-dominant PD with rest tremor of upper limbs, bradykinesia, rigidity with right-sided predominance, and postural instability; cognitive dysfunction with deficit of logical memory, visual memory, anterograde memory, and recognition capacity.
5 (24)	M/83	68	PSP-P	Atypical parkinsonian syndrome with unilateral (right) rigidity and bradykinesia, lack of tremor, and cognitive deterioration at the level of mild dementia.
6 (26)	F/85	65	PSP-P	Atypical parkinsonian syndrome with asymmetric (right) rigidity and bradykinesia, lack of tremor, and mild cognitive deficit.
7 (11/32)	F/60	46	IPD	PD with asymmetric bradykinesia and rigidity predominant on the left side, good dopaminergic responsiveness, orthostatic hypotension, depression, occasional hallucinations, and mild cognitive deficit.
8 (19/32)	M/51	46	IPD	PD with asymmetric bradykinesia and rigidity predominant on the right side, gait disorder, and good dopaminergic responsiveness.
9 (21/32)	M/48	43	IPD	PD with tremor of the left upper limb, bradykinesia, hypokinesia and rigidity with left predominance, and good dopaminergic responsiveness
10 (3/32)	F/69			Clinical data are not available

No.: number of patients, M: male, F: female, IPD: idiopathic Parkinson’s disease, MSA-C: multiple system atrophy–cerebellar phenotype; PSP-P: progressive supranuclear palsy–parkinsonism.

**Table 4:** The comparison of *LRRK2* haplotype population frequencies in the researched cohort and in other populations from 1000GP.

Number of Samples	Frequency of Haplotype
All patient samples from the study (P) n = 10 (62)	0.080
Controls from the study C1, n = 1 (69)	0.007
Controls from the study C2, n = 7 (100)	0.035
Controls from the study C3, n = 48 (2516)	0.0095
Gujarati Indian in Houston n = 11 (103)	0.053
Tuscans in Italy n = 8 (107)	0.037
Sri Lankan Tamil in the UK n = 5 (102)	0.024
Iberians in Spain n = 5 (107)	0.023
Colombians in Medellin n = 2 (94)	0.021
Puerto Rican in Puerto Rico n = 4 (104)	0.019
Bengali in Bangladesh n = 3 (86)	0.017
Punjabi in Lahore, Pakistan n = 2 (96)	0.01
Mexican Ancestry in Los Angeles, US n = 1 (64)	0.007
Finnish in Finland n = 1 (99)	0.005
Peruvian in Lima, Peru n = 1 (85)	0.005
Utah residents with Northern and Western European ancestry n = 1 (99)	0.005
African Caribbean in Barbados n = 1 (96)	0.005

n is the number of haplotype carriers; the number in brackets is the total number of the sample. Note: *LRRK2* haplotype population frequencies in our sample cohort and in other populations from 1000GP. The populations that do not have the haplotype are not shown: African ancestry in the southwestern US, Chinese Dai in Xishuangbanna (China), Han Chinese in Beijing (China), Southern Han Chinese (China), Esan in Nigeria, British in England and Scotland, Gambian in the Western Division (The Gambia), Indian Telugu in the UK, Japanese in Tokyo (Japan), Kinh in Ho Chi Minh City (Vietnam), Luhya in Webuye (Kenya), and Mende in Sierra Leone.

## Data Availability

The datasets presented in this study can be found in the online repository https://www.ncbi.nlm.nih.gov/bioproject/PRJNA682520/ (accessed on 21 December 2021).

## Supplementary Materials

The following supporting information can be downloaded at: https://www.mdpi.com/article/10.3390/life12010121/s1. Table S1. Verification of non-consanguinity: variants in HVR1 region in six random patients; Table S2. Verification of non-consanguinity: Y-STR marker region of six random patients; Table S3. Fisher’s exact factorial test (C1); Table S4. Fisher’s exact factorial test (C2); Table S5. Contingency table of chi-square test (C3); Table S6. Risk ratio (RR) and odds ratio (OR) calculation; Table S7. Presence of common LRRK2 SNPs found in the patients and 3 randomly selected controls.

## References

[1] Deng H., Gao K., Jankovic J. (2013). TheVPS35gene and Parkinson’s disease. Mov. Disord..

[2] De Lau L.M.L., Breteler M.M.B. (2006). Epidemiology of Parkinson’s disease. Lancet Neurol..

[3] Berardelli A., Rothwell J.C., Thompson P.D., Hallett M. (2001). Pathophysiology of bradykinesia in Parkinson’s disease. Brain.

[4] Noyce A., Msc J.P.B., Silveira-Moriyama L., Hawkes C.H., Giovannoni G., Lees A.J., Schrag A. (2012). Meta-analysis of early nonmotor features and risk factors for Parkinson disease. Ann. Neurol..

[5] Kieburtz K., Wunderle K.B. (2013). Parkinson’s disease: Evidence for environmental risk factors. Mov. Disord..

[6] Lill C.M. (2016). Genetics of Parkinson’s disease. Mol. Cell. Probes.

[7] Polymeropoulos M.H., Lavedan C., Leroy E., Ide S.E., Dehejia A., Dutra A., Pike B., Root H., Rubenstein J., Boyer R. (1997). Mutation in the α-Synuclein Gene Identified in Families with Parkinson’s Disease. Science.

[8] Zhang M., Cai F., Zhang S., Zhang S., Song W. (2014). Overexpression of ubiquitin carboxyl-terminal hydrolase L1 (UCHL1) delays Alzheimer’s progression in vivo. Sci. Rep..

[9] Rydning S.L., Backe P.H., Sousa M.M.L., Iqbal Z., Øye A.M., Sheng Y., Yang M., Lin X., Slupphaug G., Nordenmark T.H. (2016). NovelUCHL1 mutations reveal new insights into ubiquitin processing. Hum. Mol. Genet..

[10] Bilguvar K., Tyagi N.K., Ozkara C., Tüysüz B., Bakircioglu M., Choi M., Delil S., Caglayan A., Baranoski J.F., Erturk O. (2013). Recessive loss of function of the neuronal ubiquitin hydrolase UCHL1 leads to early-onset progressive neurodegeneration. Proc. Natl. Acad. Sci. USA.

[11] Li J.-Q., Tan L., Yu J.-T. (2014). The role of the LRRK2 gene in Parkinsonism. Mol. Neurodegener..

[12] Zimprich A., Biskup S., Leitner P., Lichtner P., Farrer M., Lincoln S., Kachergus J., Hulihan M., Uitti R.J., Calne D.B. (2004). Mutations in LRRK2 Cause Autosomal-Dominant Parkinsonism with Pleomorphic Pathology. Neuron.

[13] Greggio E., Jain S., Kingsbury A., Bandopadhyay R., Lewis P., Kaganovich A., van der Brug M.P., Beilina A., Blackinton J., Thomas K.J. (2006). Kinase activity is required for the toxic effects of mutant LRRK2/dardarin. Neurobiol. Dis..

[14] Di Maio R., Hoffman E.K., Rocha E.M., Keeney M.T., Sanders L.H., De Miranda B.R., Zharikov A., Van Laar A., Stepan A.F., Lanz T.A. (2018). LRRK2 activation in idiopathic Parkinson’s disease. Sci. Transl. Med..

[15] Ross O.A., Soto-Ortolaza A.I., Heckman M.G., Aasly J.O., Abahuni N., Annesi G., Bacon J.A., Bardien S., Bozi M., Brice A. (2011). Association of LRRK2 exonic variants with susceptibility to Parkinson’s disease: A case–control study. Lancet Neurol..

[16] Ruiz-Martínez J., Krebs C.E., Makarov V., Gorostidi A., Martí-Massó J.F., Paisán-Ruíz C. (2015). GIGYF2 mutation in late-onset Parkinson’s disease with cognitive impairment. J. Hum. Genet..

[17] Bose A., Beal M.F. (2016). Mitochondrial dysfunction in Parkinson’s disease. J. Neurochem..

[18] Mir R., Tonelli F., Lis P., Macartney T., Polinski N.K., Martinez T.N., Chou M.-Y., Howden A.J., König T., Hotzy C. (2018). The Parkinson’s disease VPS35[D620N] mutation enhances LRRK2-mediated Rab protein phosphorylation in mouse and human. Biochem. J..

[19] Chen Y.-F., Chang Y.-Y., Lan M.-Y., Chen P.-L., Lin C.-H. (2017). Identification of VPS35 p.D620N mutation-related Parkinson’s disease in a Taiwanese family with successful bilateral subthalamic nucleus deep brain stimulation: A case report and literature review. BMC Neurol..

[20] Chartier-Harlin M.-C., Dachsel J.C., Vilarino-Guell C., Lincoln S.J., Lepretre F., Hulihan M.M., Kachergus J., Milnerwood A.J., Tapia L., Song M.-S. (2011). Translation Initiator EIF4G1 Mutations in Familial Parkinson Disease. Am. J. Hum. Genet..

[21] Buervenich S., Carmine A., Galter D., Shahabi H.N., Johnels B., Holmberg B., Ahlberg J., Nissbrandt H., Eerola J., Hellström O. (2005). A Rare Truncating Mutation in ADH1C (G78Stop) Shows Significant Association With Parkinson Disease in a Large International Sample. Arch. Neurol..

[22] Gegg M.E., Schapira A.H.V. (2018). The role of glucocerebrosidase in Parkinson disease pathogenesis. FEBS J..

[23] McNeill A., Magalhaes J., Shen C., Chau K., Hughes D., Mehta A., Foltynie T., Cooper J.M., Abramov A., Gegg M. (2014). Ambroxol improves lysosomal biochemistry in glucocerebrosidase mutation-linked Parkinson disease cells. Brain.

[24] Deutschlander A.B., Konno T., Soto-Beasley A.I., Walton R.L., van Gerpen J.A., Uitti R.J., Heckman M.G., Wszolek Z.K., Ross O.A. (2020). Association of MAPT subhaplotypes with clinical and demographic features in Parkinson’s disease. Ann. Clin. Transl. Neurol..

[25] Valente E.M., Abou-Sleiman P.M., Caputo V., Muqit M.M.K., Harvey K., Gispert S., Ali Z., Del Turco D., Bentivoglio A.R., Healy D.G. (2004). Hereditary Early-Onset Parkinson’s Disease Caused by Mutations in PINK1. Science.

[26] Bonifati V., Rizzu P., van Baren M.J., Schaap O., Breedveld G.J., Krieger E., Dekker M.C.J., Squitieri F., Ibanez P., Joosse M. (2003). Mutations in the DJ-1 Gene Associated with Autosomal Recessive Early-Onset Parkinsonism. Science.

[27] Park J., Lee S.B., Lee S., Kim Y., Song S., Kim S., Bae E., Kim J., Shong M., Kim J.-M. (2006). Mitochondrial dysfunction in Drosophila PINK1 mutants is complemented by parkin. Nature.

[28] Yoshino H., Tomiyama H., Tachibana N., Ogaki K., Li Y., Funayama M., Hashimoto T., Takashima S., Hattori N. (2010). Phenotypic spectrum of patients with PLA2G6 mutation and PARK14-linked parkinsonism. Neurology.

[29] Zhao T., De Graaff E., Breedveld G.J., Loda A., Severijnen L.A., Wouters C.H., Verheijen F.W., Dekker M.C., Montagna P., Willemsen R. (2011). Loss of nuclear activity of the FBXO7 protein in patients with parkinsonian-pyramidal syn-drome (PARK15). Public Libr. Sci. One.

[30] Hirano A., Kurland L.T., Krooth R.S., Lessell S. (1961). Parkinsonism-dementia complex, an endemic disease on the island of Guam. I. Clinical features. Brain.

[31] Hirano A., Malamud N., Kurland L.T. (1961). Parkinsonism-dementia complex, an endemic disease on the island of Guam. II. Pathological features. Brain.

[32] Gajdusek D.C., Salazar A.M. (1982). Amyotrophic lateral sclerosis and parkinsonian syndromes in high incidence among the Auyu and Jakai people of West New Guinea. Neurology.

[33] De Rijk M.C., Tzourio C., Breteler M.M., Dartigues J.F., Amaducci L., Lopez-Pousa S., Manubens-Bertran J.M., Alperovitch A., Rocca W.A. (1997). Prevalence of parkinsonism and Parkinson’s disease in Europe: The EUROPARKINSON Collaborative Study. European Community Concerted Action on the Epidemiology of Parkinson’s disease. J. Neurol. Neurosurg. Psychiatry.

[34] Wermuth L., Joensen P., Bünger N., Jeune B. (1997). High prevalence of Parkinson’s disease in the Faroe Islands. Neurology.

[35] Mensikova K., Kanovsky P., Kaiserova M., Mikulicova L., Vastik M., Hlustik P., Jugas P., Ovecka J., Janout V. (2013). Prevalence of neurodegenerative parkinsonism in an isolated population in south-eastern Moravia, Czech Republic. Eur. J. Epidemiol..

[36] Bartoníková T., Menšíková K., Kolaříková K., Vodicka R., Vrtěl R., Otruba P., Kaiserová M., Vaštík M., Mikulicová L., Ovečka J. (2018). New endemic familial parkinsonism in south Moravia, Czech Republic and its genetical background. Medicine.

[37] Vodicka R., Vrtel R., Mensikova K., Kanovsky P., Dolinova I., Kolarikova K., Prochazka M. (2017). Next Generation Sequencing Data Analysis Evaluation in Patients with Parkinsonism from a Genetically Isolated Population. Genom. Comput. Biol..

[38] Menšíková K., Tučková L., Kolařiková K., Bartoníková T., Vodicka R., Ehrmann J., Vrtěl R., Procházka M., Kaňovský P., Kovacs G.G. (2019). Atypical parkinsonism of progressive supranuclear palsy–parkinsonism (PSP-P) phenotype with rare variants in FBXO7 and VPS35 genes associated with Lewy body pathology. Acta Neuropathol..

[39] Paisán-Ruíz C., Jain S., Evans E.W., Gilks W.P., Simón J., Van Der Brug M., De Munain A.L., Aparicio S., Gil A.M., Khan N. (2004). Cloning of the Gene Containing Mutations that Cause PARK8-Linked Parkinson’s Disease. Neuron.

[40] Kalia L.V., Lang A.E., Hazrati L.-N., Fujioka S., Wszolek Z.K., Dickson D.W., Ross O.A., Van Deerlin V.M., Trojanowski J.Q., Hurtig H.I. (2015). Clinical Correlations With Lewy Body Pathology inLRRK2-Related Parkinson Disease. JAMA Neurol..

[41] Schneider S.A., Alcalay R.N. (2017). Neuropathology of genetic synucleinopathies with parkinsonism: Review of the literature. Mov. Disord..

[42] Weil R.S., Lashley T., Bras J., Schrag A.E., Schott J. (2017). Current concepts and controversies in the pathogenesis of Parkinson’s disease dementia and Dementia with Lewy Bodies. F1000Research.

[43] Trinh J., Zeldenrust F.M., Huang J., Kasten M., Schaake S., Petkovic S., Madoev H., Grünewald A., Almuammar S., König I.R. (2018). Genotype-phenotype relations for the Parkinson’s disease genes SNCA, LRRK2, VPS35: MDSGene systematic review. Mov. Disord..

[44] Wallings R., Manzoni C., Bandopadhyay R. (2015). Cellular processes associated with LRRK2 function and dysfunction. FEBS J..

[45] Arranz A.M., Delbroek L., Van Kolen K., Guimaraes M.R., Mandemakers W., Daneels G., Matta S., Calafate S., Shaban H., Baatsen P. (2014). LRRK2 functions in synaptic vesicle endocytosis through a kinase-dependent mechanism. J. Cell Sci..

[46] Hakimi M., Selvanantham T., Swinton E., Padmore R.F., Tong Y., Kabbach G., Venderova K., Girardin S.E., Bulman D.E., Scherzer C.R. (2011). Parkinson’s disease-linked LRRK2 is expressed in circulating and tissue immune cells and upregulated following recognition of microbial structures. J. Neural Transm..

[47] Gardet A., Benita Y., Li C., Sands B.E., Ballester I., Stevens C., Korzenik J.R., Rioux J.D., Daly M.J., Xavier R.J. (2010). LRRK2 Is Involved in the IFN-γ Response and Host Response to Pathogens. J. Immunol..

[48] Healy D.G., Falchi M., O’Sullivan S.S., Bonifati V., Durr A., Bressman S., Brice A., Aasly J., Zabetian C.P., Goldwurm S. (2008). Phenotype, genotype, and worldwide genetic penetrance of LRRK2-associated Parkinson’s disease: A case-control study. Lancet Neurol..

[49] Lesage S., Dürr A., Tazir M., Lohmann E., Leutenegger A.-L., Janin S., Pollak P., Brice A. (2006). LRRK2G2019S as a Cause of Parkinson’s Disease in North African Arabs. N. Engl. J. Med..

[50] Ozelius L.J., Senthil G., Saunders-Pullman R., Ohmann E., Deligtisch A., Tagliati M., Hunt A.L., Klein C., Henick B., Hailpern S.M. (2006). LRRK2G2019S as a Cause of Parkinson’s Disease in Ashkenazi Jews. N. Engl. J. Med..

[51] Hui K.Y., Fernandez-Hernandez H., Hu J., Schaffner A., Pankratz N., Hsu N.-Y., Chuang L.-S., Carmi S., Villaverde N., Li X. (2018). Functional variants in the LRRK2 gene confer shared effects on risk for Crohn’s disease and Parkinson’s disease. Sci. Transl. Med..

[52] Xiao B., Deng X., Ng E.Y.-L., Allen J.C., Lim S.-Y., Ahmad-Annuar A., Tan E.-K. (2018). Association of LRRK2 Haplotype with Age at Onset in Parkinson Disease. JAMA Neurol..

[53] Heckman M.G., Elbaz A., Soto-Ortolaza A.I., Serie D.J., Aasly J.O., Annesi G., Auburger G., Bacon J.A., Boczarska-Jedynak M., Bozi M. (2013). The protective effect of LRRK2 p.R1398H on risk of Parkinson’s disease is independent of MAPT and SNCA variants. Neurobiol. Aging.

[54] Paisã¡n-Ruiz C. (2009). LRRK2gene variation and its contribution to Parkinson disease. Hum. Mutat..

[55] Paisán-Ruiz C., Washecka N., Nath P., Singleton A.B., Corder E.H. (2009). Parkinson’s Disease and Low Frequency Alleles Found Together ThroughoutLRRK2. Ann. Hum. Genet..

[56] Setó-Salvia N., Clarimón J., Pagonabarraga J., Pascual-Sedano B., Campolongo A., Combarros O., Mateo J.I., Regaña D., Martínez-Corral M., Marquié M. (2011). Dementia Risk in Parkinson Disease. Arch. Neurol..

[57] Smith W.W., Pei Z., Jiang H., Dawson V.L., Dawson T.M., A Ross C. (2006). Kinase activity of mutant LRRK2 mediates neuronal toxicity. Nat. Neurosci..

[58] Gilsbach B.K., Ekortholt A. (2014). Structural biology of the LRRK2 GTPase and kinase domains: Implications for regulation. Front. Mol. Neurosci..

[59] Auton A., Brooks L.D., Durbin R.M., Garrison E.P., Kang H.M., Korbel J.O., Marchini J.L., McCarthy S., McVean G.A., 1000 Genomes Project Consortium (2015). A global reference for human genetic variation. Nature.

[60] Greggio E., Cookson M.R. (2009). Leucine-Rich Repeat Kinase 2 Mutations and Parkinson’s Disease: Three Questions. ASN Neuro.

[61] Deng X., Dzamko N., Prescott A., Davies P., Liu Q., Yang Q., Lee J.-D., Patricelli M.P., Nomanbhoy T.K., Alessi D.R. (2011). Characterization of a selective inhibitor of the Parkinson’s disease kinase LRRK2. Nat. Chem. Biol..

