# Supplementary material for: High-Throughput Sequencing Haplotype Analysis Indicates in LRRK2 Gene a Potential Risk Factor for Endemic Parkinsonism in Southeastern Moravia, Czech Republic
Source: Life (Basel). 2022 Jan 14;12(1):121. doi: 10.3390/life12010121 (PMC8780375; doi:10.3390/life12010121)
Supplement: Supplementary file 1 [file life-12-00121-s001.zip › life-1542325 supplementary.pdf]

Article

High-Throughput Sequencing Haplotype Analysis Indicates in *LRRK2* Gene a Potential Risk Factor for Endemic Parkinsonism in Southeastern Moravia, Czech Republic

Kristyna Kolarikova [1,2,3,4], Radek Vodicka [1,2,*], Radek Vrtel [1,2], Julia Stellmachova [1,2], Martin Prochazka [1,2], Katerina Mensikova [3,4], Tereza Bartonikova [3,4], Tomas Furst [5], Petr Kanovsky [3,4] and Jan Geryk [6,7]

Supplementary Material

Table S1. Verification of non-consanguinity: variants in HVR1 region in six random patients.

	16093C	16113C	16126C	16129A	16172C	16223T	16224C	16270A	16294T	16296T	16304C	16311C	16391A	16465A	16519C	Haplogroup
Patient 1 (3)																HV*
Patient 2 (17)																HV*
Patient 3 (22)																K
Patient 4 (23)																T
Patient 5 (24)																U*
Patient 6 (26)																I

The green highlighted cells indicate the presence of the variant.

Table S2. Verification of non-consanguinity: Y-STR marker region of six random patients.

	DYS19	DYS385a/b	DYS389I/II	DYS390	DYS391	DYS392	DYS393	DYS437	438	439	DYS448	DYS456	DYS458	C4	H4
**Patient 1 (3)**	14	13/17	13/29	23	11	11	12	15	9	11	20	15	16	21	10
**Patient 2 (17)**	13	17/18	14/31	25	11	11	14	14	10	11	18	15	15	22	12
**Patient 3 (22)**	17	11/14	13/30	26	11	11	13	14	11	12	20	16	17	23	12
**Patient 4 (23)**	17	11/14	13/31	25	11	11	13	14	11	10	20	15	14	23	14
**Patient 5 (24)**	14	11/14	14/30	24	10	13	12	15	12	12	19	16	15	23	11
**Patient 6 (26) female**															

Table S3. Fisher's exact factorial test (C1).

	PAR	nonPAR
H	10	1
nonH	52	68

p-value 0.0031; H – number of patients with haplotype, nonH – number of patients without haplotype, PAR – number of PD patients, nonPAR – number of healthy controls.

Table S4. Fisher's exact factorial test (C2).

	PAR	nonPAR
H	10	7
nonH	52	93

p-value 0.11; H – number of patients with haplotype, nonH – number of patients without haplotype, PAR – number of PD patients, nonPAR – healthy controls.

Table S5. Contingency table of chi-square test (C3).

	PAR	nonPAR	total
H	10+1	47	10 +48
nonH	52 +p*2516-1	(1-p)*2516-47	62+2516-48
total	62+p*2516	(1-p)*2516	62+2516

p-value < 0.00001; H – number of patients with haplotype, nonH – number of patients without haplotype, PAR – number of PD patients, nonPAR – healthy controls, p – prevalence of PD in the population (assumed p=0.01). Haplotype frequency and RR and OR calculation (we assume p=0.01 i.e. one in a hundred in the control population has PAR):

Table S6. Risk ratio (RR) and odds ratio (OR) calculation.

	C1	C2	C3
**RR**	2.09	1.65	6.3
**OR**	13.15	2.58	7.6

C1 is study group of 69 age-matched controls from the researched area; C2 is study group of 100 healthy non-related individuals from the Czech population; C3 is study group of 2516 samples from the 1000 Genome Project.

Table S7. Presence of common LRRK2 SNPs found in the patients and 3 randomly selected controls.

Coordinate (hg19)	ref	Patients LRRK2 genotypes						Controls LRRK2 genotypes			Estimated patients LRRK2 haplotype
**chr12:40626230**	**T**	**T/C**	**T/C**	**T/C**	**T/C**	**T/C**	T/C	T/C	T/C	T/C	C
chr12:40631791	T	T/T	T/C	T/C	T/C	T/C	T/C	T/C	T/C	C/C	T
chr12:40634203	A	A/G	A/G	A/G	A/G	A/G	A/G	A/A	A/A	A/A	A/G
chr12:40645257	C	C/C	C/A	C/C	C/C	C/C	C/A	C/A	C/C	C/C	C
chr12:40677655	C	C/T	C/T	C/T	C/T	C/T	C/T	C/C	C/C	C/C	T
chr12:40681142	C	C/C	C/CT	C/C	C/CT	C/CT	C/CT	C/CT	C/CT	CT/CT	C
chr12:40688695	T	T/C	T/T	T/T	T/T	T/T	T/T	T/T	T/T	T/T	T
chr12:40702846	A	A/T	T/T	A/T	T/T	T/T	T/T	T/T	A/T	T/T	T
chr12:40703046	CT	CT/C	CT/C	CT/C	CT/C	CT/C	CT/C	CT/CT	CT/CT	CT/CT	CT/C
chr12:40703087	C	C/CGT	C/CGT	C/CGT	C/CGT	C/CGT	C/CGT	C/CGT	C/CGT	C/CGT	C/CGT
chr12:40707861	C	C/C	C/C	C/C	C/C	C/C	C/C	C/T	C/C	C/C	C
chr12:40708892	T	T/C	C/C	T/C	C/C	C/C	C/C	C/C	T/C	C/C	C
chr12:40708922	C	C/T	T/T	C/T	T/T	T/T	T/T	C/T	C/C	T/T	T
chr12:40713759	T	T/C	C/C	T/C	C/C	C/C	C/C	T/C	T/T	C/C	C
chr12:40713834	C	C/A	A/A	C/A	A/A	A/A	A/A	A/A	C/A	A/A	A
chr12:40713873	A	A/G	G/G	A/G	A/G	G/G	A/G	A/G	A/A	A/G	G
chr12:40713901	T	T/T	T/A	T/T	T/T	T/T	T/A	T/A	T/T	T/T	T
chr12:40714009	A	A/A	A/A	A/A	A/A	A/G	A/A	A/A	A/G	A/A	A
chr12:40716015	C	C/T	T/T	C/T	C/T	C/T	T/T	T/T	C/T	T/T	T
chr12:40716260	T	T/C	C/C	T/C	C/C	C/C	C/C	T/C	T/T	C/C	C
chr12:40740686	A	A/G	A/G	A/G	A/G	A/G	A/G	A/A	A/A	A/A	A/G
chr12:40742254	G	G/G	G/A	A/G	A/G	G/G	G/G	G/G	G/G	G/A	G
chr12:40757330	A	A/A	A/A	A/A	A/A	A/A	A/G	G/G	A/G	A/A	A
chr12:40758652	T	T/T	T/C	T/T	T/C	T/C	T/C	C/C	C/C	C/C	T
chr12:40760764	T	T/C	T/C	T/C	T/C	T/C	T/C	T/T	T/T	T/T	T/C

The yellow highlighted cells indicates the different allele than is present in potentially risk haplotype.
